# Supplementary material for: Immunostimulatory Effects of Guanine-Quadruplex Topologies as Scaffolds for CpG Oligodeoxynucleotides
Source: Biomolecules. 2025 Jan 10;15(1):95. doi: 10.3390/biom15010095 (PMC11763011; doi:10.3390/biom15010095)
Supplement: Supplementary file 1 [file biomolecules-15-00095-s001.zip › biomolecules-3345338-supplementary.pdf]

Supporting Information

Immunostimulatory Effects of Guanine-Quadruplex Topologies as Scaffolds for CpG Oligodeoxynucleotides

Soumitra Pathak [1,2], Nguyen Bui Thao Le [1,2], Taiji Oyama [3,4], Yusuke Odahara [5], Atsuya Momotake [5], Kazunori Ikebukuro [4], Chiho Kataoka-Hamai [1], Chiaki Yoshikawa [1,2], Kohsaku Kawakami [1], Yoshihisa Kaizuka [1] and Tomohiko Yamazaki [1,2,*]

[1] Research Center for Macromolecules and Biomaterials, National Institute for Materials Science (NIMS), 1-2-1 Sengen, Tsukuba 305-0047, Japan; pathak.soumitra@nims.go.jp (S.P.); jtef1940@tmd.ac.jp (N.B.T.L.); kataoka.chiho@nims.go.jp (C.K.-H.); yoshikawa.chiaki@nims.go.jp (C.Y.); kawakami.kohsaku@nims.go.jp (K.K.); kaizuka.yoshihisa@nims.go.jp (Y.K.)

[2] Graduate School of Life Science, Hokkaido University, Kita 10, Nishi 8, Sapporo 060-0808, Japan

[3] JASCO Corporation, Hachioji 192-8537, Japan; taiji.oyama@jasco.co.jp

[4] Department of Biotechnology and Life Science, Graduate School of Engineering, Tokyo University of Agriculture and Technology, Koganei 184-8588, Japan; ikebu@cc.tuat.ac.jp

[5] Department of Chemistry, University of Tsukuba, Tsukuba 305-8571, Japan; s2220202@u.tsukuba.ac.jp (Y.O.); amomotak@chem.tsukuba.ac.jp (A.M.)

*** Corresponding authors:**

Tomohiko Yamazaki (T.Y.), Email: yamazaki.tomohiko@nims.go.jp

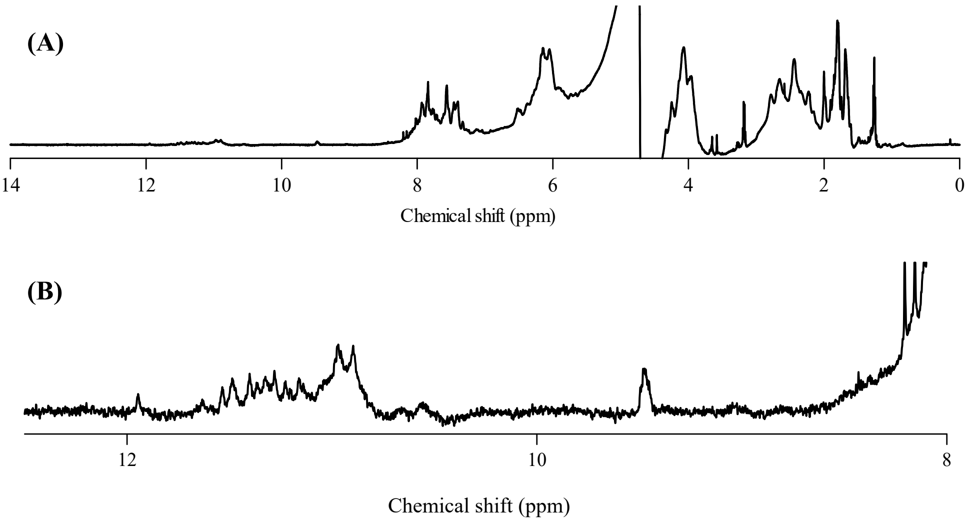

Supplementary Figure S1. ^{1}H NMR spectra of GD2_P, (A) the full scale and (B) only the imino proton region.

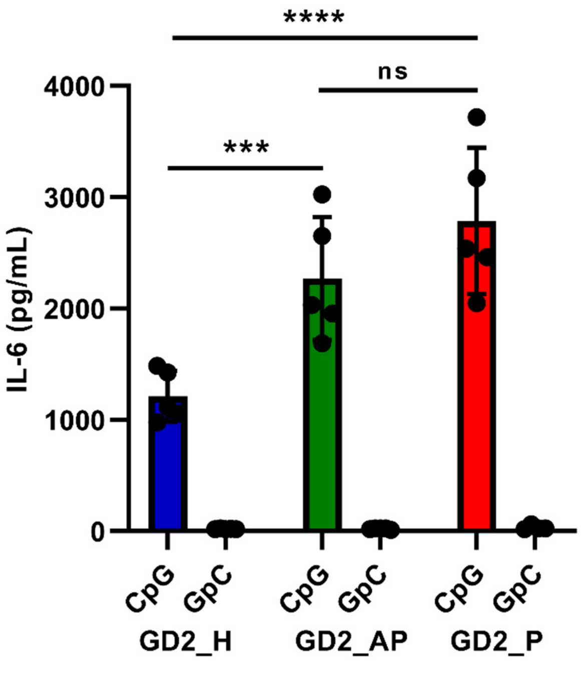

Supplementary Figure S2. A comparison of cytokine induction between G4 CpG ODNs and G4 GpC ODNs after incubating them in mouse macrophage RAW cells for 24h. D-PBS was used as control. In the figure, ****p < 0.0001 , ***p < 0.001 and ^{ns}p ≥ 0.05 (two-way ANOVA, Tukey's multiple comparisons test for comparison with other groups).

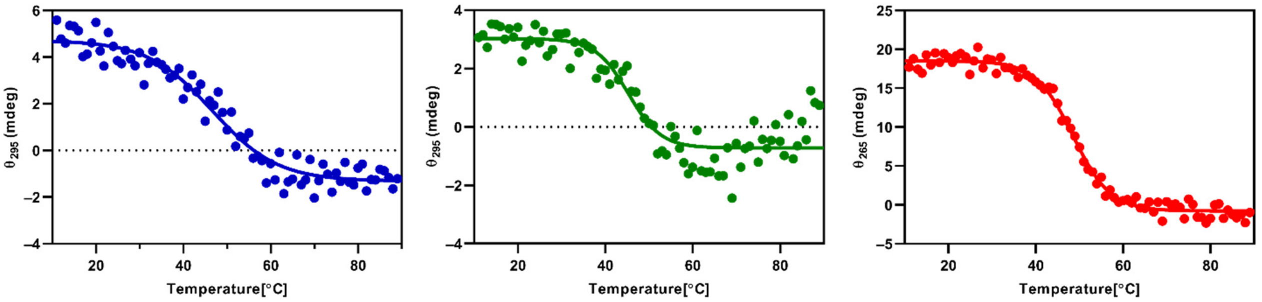

Supplementary Figure S3. Melting curve analysis of G4 CpG ODNs by heating the G4 CpG ODNs from 10°C to 90°C in DPBS buffer. GD2_H and GD2_AP was scanned at 295 nm and GD2_P was scanned at 265 nm.

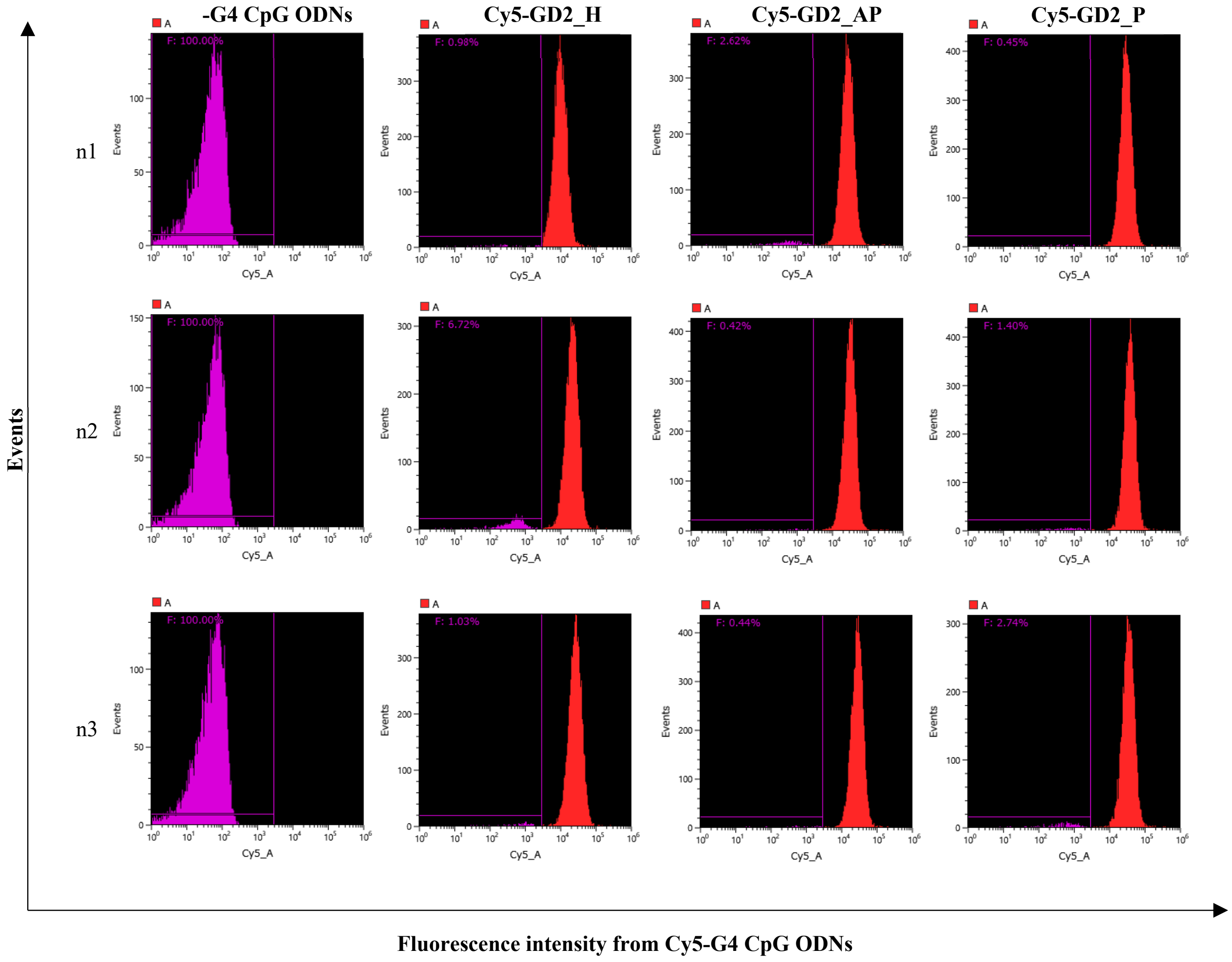

Supplementary Figure S4. Histogram analysis of fluorescence intensity from RAW264 cells treated with Cy5 labeled G4 CpG ODNs.

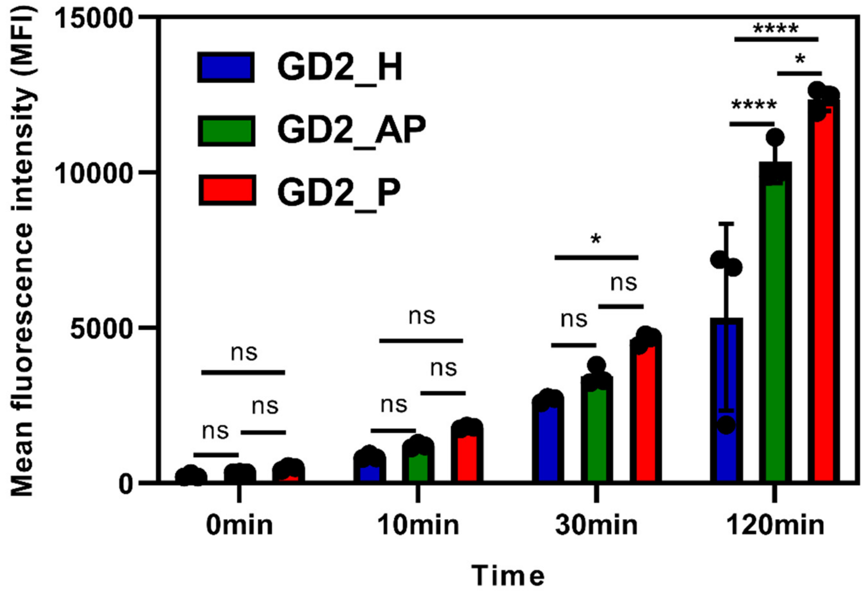

Supplementary Figure S5. Comparison of rate of cellular uptake of Cy5 labeled G4 CpG ODNs by RAW cells after incubating for 2h. Mean fluorescence intensity was measured using flow cytometry. In the figure, ****p < 0.0001 , *p < 0.05 and ^{ns}p ≥ 0.05 (two-way ANOVA, Tukey's multiple comparisons test for comparison with other groups).

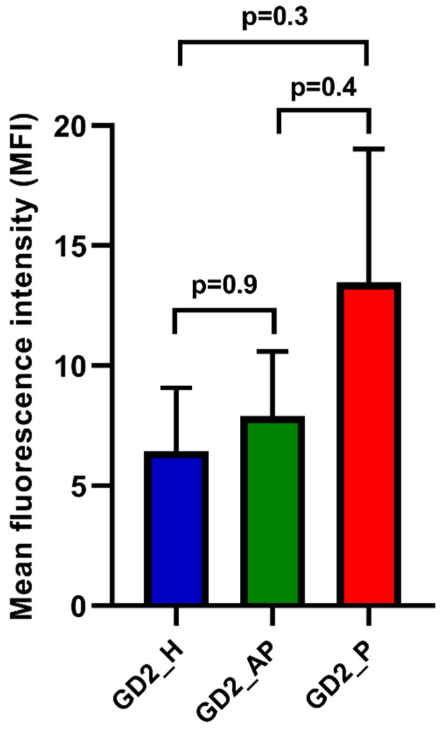

Supplementary Figure S6. Internalization of Cy5 labeled G4 CpG ODNs in RAW cells. The quantification was done using Fiji (ImageJ). In the graph, p values were calculated based on a one-way ANOVA, Tukey's multiple comparisons test for comparison with other groups.

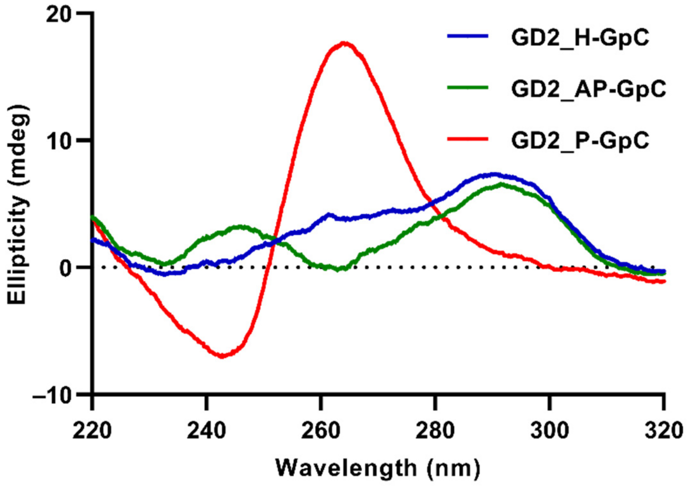

Supplementary Figure S7. Circular dichroism (CD) spectrum of G4 GpC ODNs in DPBS at 25°C.

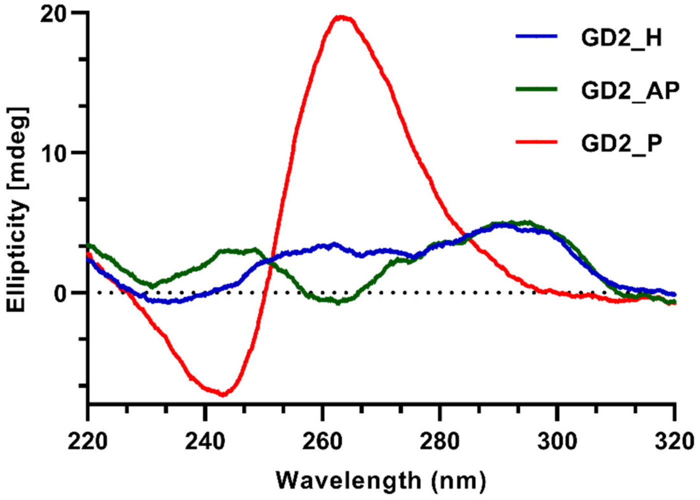

Supplementary Figure S8. Circular dichroism (CD) spectrum of G4 CpG ODNs in DPBS at 25°C.
